# Supplementary material for: Measurement-induced collective vibrational quantum coherence under spontaneous Raman scattering in a liquid
Source: Nat Commun. 2023 May 17;14:2818. doi: 10.1038/s41467-023-38483-9 (PMC10192212; doi:10.1038/s41467-023-38483-9)
Supplement: Supplementary file 1 — Supplementary Information [file 41467_2023_38483_MOESM1_ESM.pdf]

Supplementary Information

Measurement-Induced Collective Vibrational Quantum Coherence under Spontaneous Raman Scattering in a Liquid

Valeria Vento,[1,*] Santiago Tarrago Velez,[1,*] Anna Pogrebna,[1] and Christophe Galland[1,†]

[1]*École polytechnique fédérale de Lausanne (EPFL), Institute of Physics, CH-1015 Lausanne, Switzerland*

(Dated: April 20, 2023)

Supplementary Notes

Full quantum model.— The four collective vibrational modes of interest in our experiment are described by the annihilation operators $\hat{b}_1$, $\hat{b}_2$, $\hat{b}_3$, $\hat{b}_4$. Under sufficiently short excitation pulse and single spatial mode filtering, all four vibrational modes effectively couple to the same Stokes and anti-Stokes photon fields, described by the annihilation operators $\hat{a}_S^x$, $\hat{a}_A^x$, where $x = w, r$ for the write and read pulses, respectively. The Raman interaction is modeled by the Hamiltonian [1]

$$\begin{aligned}\hat{H}_I^x = \hbar\alpha^x \Big[\lambda_S^x(\hat{a}_S^x)^\dagger(\beta_1\hat{b}_1^\dagger + e^{-i\theta_{2,S}^x}\beta_2\hat{b}_2^\dagger \\ + e^{-i\theta_{3,S}^x}\beta_3\hat{b}_3^\dagger + e^{-i\theta_{4,S}^x}\beta_4\hat{b}_4^\dagger) \\ + \lambda_A^x(\hat{a}_A^x)^\dagger(\beta_1\hat{b}_1 + e^{-i\theta_{2,A}^x}\beta_2\hat{b}_2 \\ + e^{-i\theta_{3,A}^x}\beta_3\hat{b}_3 + e^{-i\theta_{4,A}^x}\beta_4\hat{b}_4)\Big] + h.c.\end{aligned} \tag{1}$$

where the laser is modeled by a coherent field of amplitude $\alpha_x(t)$ $(x = w, r)$ with Gaussian envelope centered at time t_{0x} of width σ_x,

$$\alpha^x(t) = A_x \exp\left(\frac{(t - t_{0x})^2}{2\sigma_x^2}\right)\exp\left(-i\omega_x t\right). \tag{2}$$

In eq. (1) $\lambda_{S,A}^x$ determine the coupling strengths to the Stokes and anti-Stokes modes, and the β_i's are real numbers satisfying $\sum_i \beta_i^2 = 1$ where β_i^2 control the relative intensity of the Raman peak corresponding to vibrational mode i, experimentally determined by the relative isotopic abundance and the temperature.

Since we use spectral filtering and post-selection to ignore events where an anti-Stokes (resp. Stokes) photon is emitted during the write (resp. read) pulse we can simplify the interaction model to

$$\hat{H}_I^w = \hbar\lambda_S^w\alpha^w(\hat{a}_S^w)^\dagger(\beta_1\hat{b}_1^\dagger + e^{-i\theta_{2,S}^w}\beta_2\hat{b}_2^\dagger \tag{3}$$

$$+ e^{-i\theta_{3,S}^w}\beta_3\hat{b}_3^\dagger + e^{-i\theta_{4,S}^w}\beta_4\hat{b}_4^\dagger) + h.c. \tag{4}$$

$$\hat{H}_I^r = \hbar\lambda_A^r\alpha^r(\hat{a}_A^r)^\dagger(\beta_1\hat{b}_1 + e^{-i\theta_{2,A}^r}\beta_2\hat{b}_2 \tag{5}$$

$$+ e^{-i\theta_{3,A}^r}\beta_3\hat{b}_3 + e^{-i\theta_{4,A}^r}\beta_4\hat{b}_4) + h.c. \tag{6}$$

(Note that the ignored terms contribute to uncorrelated noise photons generated via higher order Raman interactions during a single pulse [2]. Such noise is accounted for below.)

We have explicitly written the phase differences $\theta_{i,S}^w$, resp. $\theta_{i,A}^r$, appearing between the three vibrational modes $(i = 2, 3, 4)$ during Stokes, resp. anti-Stokes, scattering, taking the global phase such that $\theta_1^{w,r} = 0$. The experiment is however only sensitive to the sums $\theta_i = \theta_{i,S}^w + \theta_{i,A}^r$. Interestingly, we find excellent fit of the data for $\theta_i = 0$ for $i = 2, 3, 4$, which we tentatively relate to the very large detuning between the laser pulses and any electronic resonance of CS_2. In the following we shorten the notation for the annihilation operators to $\hat{a}_S^w \equiv \hat{a}_S$ and $\hat{a}_A^r \equiv \hat{a}_A$.

We include an additional $\chi^{(3)}$ nonlinear interaction term that allows for the direct interaction between the write and read pulses, leading to the creation of photon pairs at the frequencies of the Stokes and anti-Stokes emission (FWM process):

$$\hat{H}_I^{(3)} = \hbar\lambda^{(3)}\alpha^w\alpha^r\hat{a}_S\hat{a}_A + h.c. \tag{7}$$

In the frame rotating with the central frequency $\omega_0 = \frac{\omega_w + \omega_r}{2}$ we obtain the effective Hamiltonian

$$\begin{aligned}\hat{H} = \hbar\Omega_1\hat{b}_1^\dagger\hat{b}_1 + \hbar\Omega_2\hat{b}_2^\dagger\hat{b}_2 + \hbar\Omega_3\hat{b}_3^\dagger\hat{b}_3 + \hbar\Omega_4\hat{b}_4^\dagger\hat{b}_4 \\ + \hbar\Delta_S\hat{a}_S^\dagger\hat{a}_S + \hbar\Delta_A\hat{a}_A^\dagger\hat{a}_A \\ + \hat{H}_I^w + \hat{H}_I^r + \hat{H}_I^{(3)}\end{aligned} \tag{8}$$

where $\Delta_{S,A} = \omega_{S,A} - \omega_0$. To account for dissipation we use the master equation approach, which includes coupling of the phonon modes to a thermal reservoir at room temperature, as described by the collapse operators

$$\begin{aligned}\hat{C}_{bi-} = \sqrt{\kappa_i(1 + n_{th})}\hat{b}_i \\ \hat{C}_{bi+} = \sqrt{\kappa_i n_{th}}\hat{b}_i^\dagger\end{aligned} \tag{9}$$

where $i = 1, 2, 3, 4$ and κ_i is related to the decay rate of the phonon modes by $\tau_{ph_i} = 1/\kappa_i$. The temporal evolution of the density matrix is computed numerically using QuTiP, an open-source library used for simulating quantum systems in Python [3, 4].

Coincidence counts under non-ideal conditions.— To account for noise and losses in the experiment we use the

* These authors contributed equally to this work
† chris.galland@epfl.ch

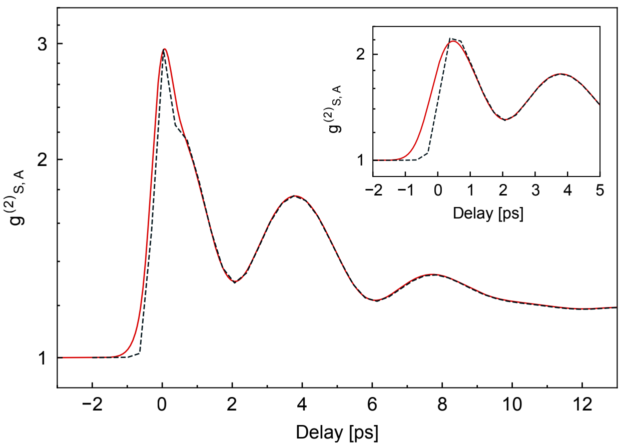

Supplementary Figure S1. **Model comparison.** Comparison between the full quantum model (dashed black line) and the analytical model used in the main text to fit the experimental data (solid red line). In the inset, the two models are shown with FWM hamiltonian $\hat{H}_I^{(3)} = 0$.

operator introduced in [5] to model the photon detection probability

$$\hat{D}_X = 1 - (1 - p_X^{dc})(1 - \eta_X)^{\hat{a}_X^\dagger \hat{a}_X} \quad (10)$$

where $X = S, A$ for the Stokes and anti-Stokes detection channels, respectively. The dark count probability (per detection time window) is p_X^{dc} while η_X is the detection efficiency. The experimental value of the normalised Stokes – anti-Stokes coincidence rate $g_{S,A}^{(2)}$ is then calculated (for several values of $t = t_{0r} - t_{0w}$) as

$$g_{S,A}^{(2)} = \frac{\left\langle \hat{D}_S \hat{D}_A \right\rangle}{\left\langle \hat{D}_S \right\rangle \left\langle \hat{D}_A \right\rangle}. \quad (11)$$

Comparison with the analytical model.— In order to corroborate the quality of the analytical model that we presented in the main text, we used the full quantum model with the analytically-extracted parameters to simulate our experimental results. Therefore, we set $\theta_2 = \theta_3 = \theta_4 = 0$; $\Omega_i/(2\pi) \simeq 20.04$, 19.96, 19.81 and 19.76 THz and $\tau_{ph_i} = T_i/2$ (see Ref.[6]) with $T_i \simeq 15.71$, 18.38, 6.77 and 15.71 ps as extracted from the cw spectrum, and the weights $\beta_i^2 \simeq 0.70$, 0.03, 0.21 and 0.06.

The detection efficiency of our setup is estimated to be $\eta_S \approx \eta_A \approx 10\%$. We fix the values of the parameters A_x and $\lambda_{S,A}^x$ by considering the Stokes and anti-Stokes detection rates. As the pulse amplitudes A_x always appear in factor with the coupling rates λ^x, we introduce $\Lambda_{S,A}^x = A_x \lambda_{S,A}^x$ whose values are chosen to reproduce the measured single-detector count rate when only the write or read pulse is propagating through the sample. We find $\Lambda_S^w = 0.102$ and $\Lambda_A^r = 0.131$, which recover the measured detection probabilities $p_S \approx 2.5 \times 10^{-4}$, $p_A \approx 1.6 \times 10^{-5}$. The dark count probabilities are estimated to be $p_S^{dc} \approx 2 \times 10^{-4}$ and $p_A^{dc} \approx 1 \times 10^{-5}$ and include

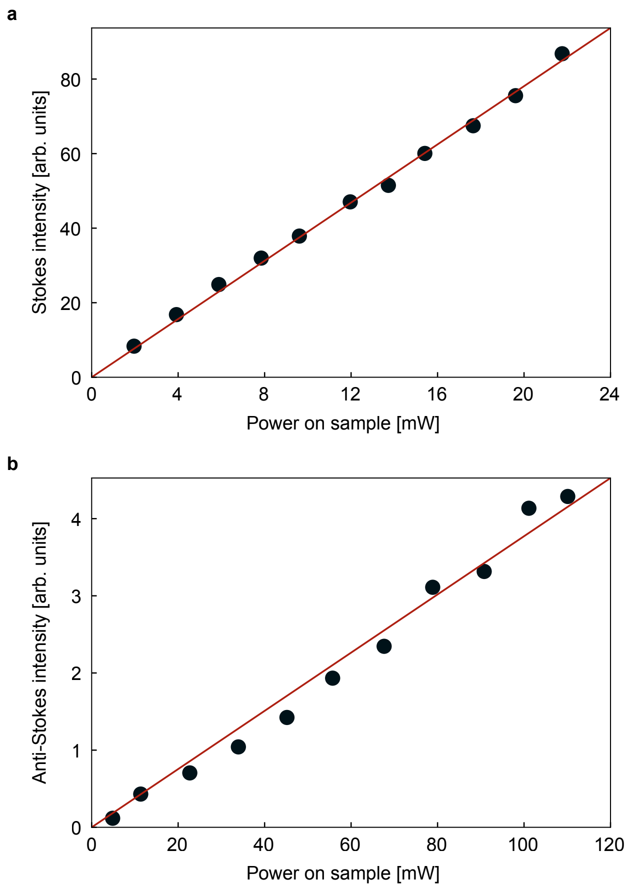

Supplementary Figure S2. **Power dependence of the Stokes and anti-Stokes intensities. a** Stokes intensity as a function of the write power on the sample. **b** Anti-Stokes intensity as a function of the read power on the sample. The counts are integrated over the relevant range of the Raman spectrum corresponding to the correlation measurements. The red lines are linear regressions. In the photon counting experiment, the time-averaged laser powers are below 7 and 45 mW for write and read beams, respectively – well within the linear regime of spontaneous Raman scattering.

detector noise plus background emission noise. The coupling rate accounting for all the FWM processes at zero delay has been set to $\Lambda_{FWM} = A_w A_r \lambda^{(3)} = 0.0094$ in order to reproduce the experimental results.

Figure S1 shows the match between the two models, where the discrepancy around zero delay is attributed to additional dispersion in the optics that increases the rise time (not considered in the quantum model).

Supplementary Methods

Power dependence.— For each measurement, the time-averaged write power on the sample is in the range [5-7] mW, while the time-averaged read power is in the range [35-45] mW, both at 80 MHz repetition rate (1 mW

corresponds to 12.5 pJ pulse energy). We measured for different powers the Stokes intensity generated by the write pulse, as well as the anti-Stokes intensity generated by the read pulse (which is close in frequency to the write pulse, so no significant difference in Raman cross section is expected). The integrated Stokes and anti-Stokes counts are plotted in Figs. S2a and S2b respectively. The power dependence is best fit by a linear regression for both of them and shows no sign of quadratic increase, excluding a significant contribution of stimulated Raman scattering.

In addition, these data put a stringent upper bound on the mean number of Stokes photons generated by pulse (in the spatial mode we collect). Indeed, for the anti-Stokes signal, the crossover from linear to quadratic power dependence occurs when the mean Stokes photon number (and therefore generated phonon number) per pulse is on the order of the thermal occupancy [7, 8], i.e. 0.04. We find no sign of quadratic increase up to 100 mW pump power. Therefore, in the conditions of the correlation measurements (~ 5 mW write power), the mean excited phonon number is expected to be on the order of 2×10^{-3}.

Detailed setup and detection probabilities.— A detailed scheme of the experimental setup is given in Fig. S3a, showing in particular the layout of the spectral filters used in excitation and detection. The acquired spectra of the write and read pulses are displayed in Figs. S3a-b, and present FWHM around 80 cm^{-1} and 190 cm^{-1} respectively. Therefore, each pulse linewidth is at least 3 times smaller than the frequency of Raman active modes around 655 cm^{-1}, which excludes the possibility of generating coherence with a single pulse, i.e. through impulsive stimulated Raman scattering. Notice also that any tail potentially overlapping with the Raman signal is filtered before impinging on the sample.

Furthermore, we wish to exclude any hypothetical form of classical coherent oscillation arising from an eventual low-frequency resonance excited by the first pulse and modulating the probability of collecting an anti-Stokes photon from the second pulse. In Fig. S4 we report several consecutive measurements of the Stokes and anti-Stokes detection probabilities as a function of the write-read delay. Contrary to the two-photon coincidence measurements shown in the main text, the single-photon data show no relevant signal and are dominated by laser power fluctuations. Note that, in presence of classical coherence, the second-order correlation function measured in the main text would actually show no oscillations: since the Stokes detection probability doesn't depend on the induced coherence probed by the anti-Stokes signal, the numerator in Eq. 11 factorizes and, as a consequence, it results $g^{(2)}_{S,A} = 1$, whatever the detailed mechanisms at play. This discussion highlights how second-order correlation functions can robustly and unambiguously pinpoint quantum v.s classical coherence in optics, as originally established by Roy J. Glauber and co-workers (cf. Nobel Prize 2005).

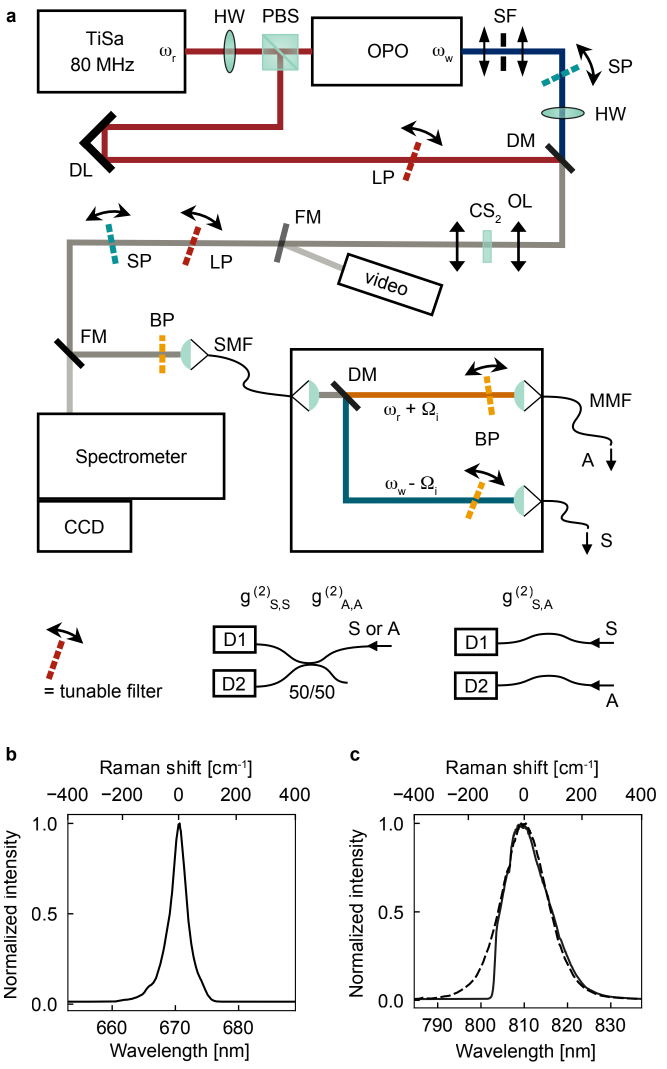

Supplementary Figure S3. **Detailed experimental setup. a** Detailed schematics of the experimental setup. HW: half-wave plate, DL: delay line, PBS: polarizing beam splitter, DM: dichroic mirror, SF: spatial filter, SP: short-pass filter, LP: long-pass filter, BP: band-pass filter, OL: objective lens, FM: flip mirror, SMF: single-mode fiber, MMF: multimode fiber, S: Stokes, A: anti-Stokes; D1, D2, D3, D4: detectors. **b** Write and **c** read pulses measured with the spectrometer. The dashed line is measured after removing the LP filter in the Ti:Sa path in order to estimate an upper bound for the pulse width.

Supplementary references

[1] T. von Foerester and R. J. Glauber, Quantum theory of light propagation in amplifying media, Phys. Rev. A **3**, 1484 (1971).

[2] C. A. Parra-Murillo, M. F. Santos, C. H. Monken, and A. Jorio, Stokes–anti-stokes correlation in the inelastic scattering of light by matter and generalization of the bose-einstein population function, Phys. Rev. B **93**, 125141 (2016).

[3] J. R. Johansson, P. D. Nation, and F. Nori, Qutip:

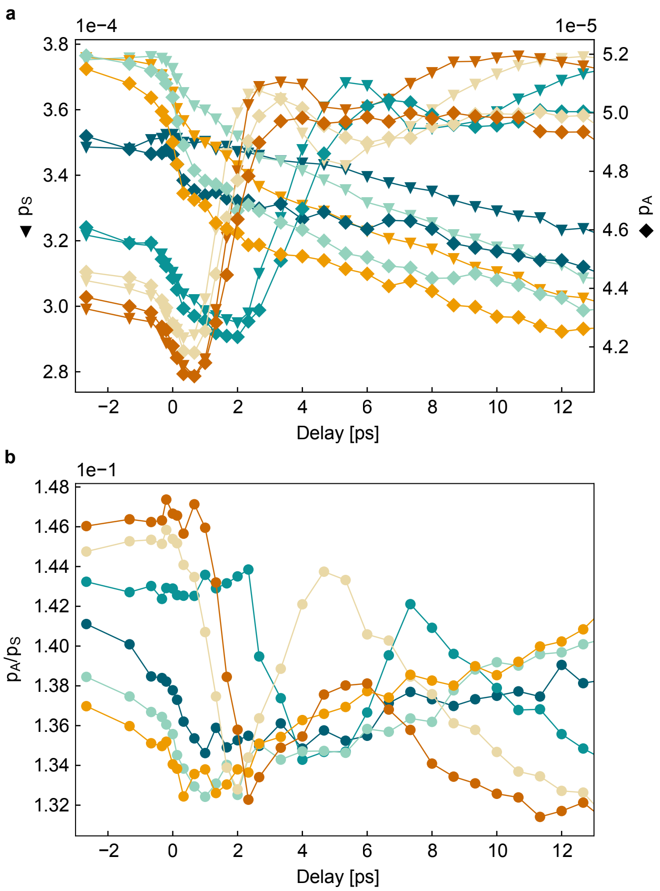

Supplementary Figure S4. **Time fluctuations of the detection probability. a** Stokes (triangles) and anti-Stokes (squares) detection probabilities and **b** Stokes-anti-Stokes probability ratio as a function of the write-read delay for different consecutive measurements (indicated by different colours). The time steps are the same employed in the $g^{(2)}$ measurements presented in the main text, where a higher density of points is acquired around zero delay, explaining the larger impact of laser power drifts in this region.

An open-source python framework for the dynamics of open quantum systems, Computer Physics Communications **183**, 1760 (2012).

[4] J. R. Johansson, P. D. Nation, and F. Nori, Qutip 2: A python framework for the dynamics of open quantum systems, Comp. Phys. Comm **184**, 1234 (2013).

[5] P. Sekatski, N. Sangouard, F. Bussieres, C. Clausen, N. Gisin, and H. Zbinden, Detector imperfections in photon-pair source characterization, J. Phys. B At. Mol. Opt. Phys. **45**, 124016 (2012).

[6] S. T. Velez, K. Seibold, N. Kipfer, M. D. Anderson, V. Sudhir, and C. Galland, Preparation and Decay of a Single Quantum of Vibration at Ambient Conditions, Physical Review X **9**, 41007 (2019).

[7] M. K. Schmidt, R. Esteban, F. Benz, J. J. Baumberg, and J. Aizpurua, Linking classical and molecular optomechanics descriptions of SERS, Faraday Discussions **205**, 31 (2017).

[8] M. K. Schmidt, R. Esteban, A. González-Tudela, G. Giedke, and J. Aizpurua, Quantum Mechanical Description of Raman Scattering from Molecules in Plasmonic Cavities, ACS Nano **10**, 6291 (2016).
